# Immune Cell Profiling During Switching from Natalizumab to Fingolimod Reveals Differential Effects on Systemic Immune-Regulatory Networks and on Trafficking of Non-T Cell Populations into the Cerebrospinal Fluid—Results from the ToFingo Successor Study

**DOI:** 10.3389/fimmu.2018.01560

**Published:** 2018-07-09

**Authors:** Lisa Lohmann, Claudia Janoschka, Andreas Schulte-Mecklenbeck, Svenja Klinsing, Lucienne Kirstein, Uta Hanning, Timo Wirth, Tilman Schneider-Hohendorf, Nicholas Schwab, Catharina C. Gross, Maria Eveslage, Sven G. Meuth, Heinz Wiendl, Luisa Klotz

**Affiliations:** ^1^Department of Neurology with Institute of Translational Neurology, University Hospital Münster, Muenster, Germany; ^2^Department of Radiology, University Hospital Münster, Muenster, Germany; ^3^Institute of Biostatistics and Clinical Research, University of Münster, Muenster, Germany

**Keywords:** multiple sclerosis, cerebrospinal fluid, natalizumab, fingolimod, viSNE, spanning-tree progression analysis of density-normalized events, immunoregulatory network

## Abstract

Leukocyte sequestration is an established therapeutic concept in multiple sclerosis (MS) as represented by the trafficking drugs natalizumab (NAT) and fingolimod (FTY). However, the precise consequences of targeting immune cell trafficking for immunoregulatory network functions are only incompletely understood. In the present study, we performed an in-depth longitudinal characterization of functional and phenotypic immune signatures in peripheral blood (PB) and cerebrospinal fluid (CSF) of 15 MS patients during switching from long-term NAT to FTY treatment after a defined 8-week washout period within a clinical trial (ToFingo successor study; ClinicalTrials.gov: NCT02325440). Unbiased visualization and analysis of high-dimensional single cell flow-cytometry data revealed that switching resulted in a profound alteration of more than 80% of investigated innate and adaptive immune cell subpopulations in the PB, revealing an unexpectedly broad effect of trafficking drugs on peripheral immune signatures. Longitudinal CSF analysis demonstrated that NAT and FTY both reduced T cell subset counts and proportions in the CSF of MS patients with equal potency; NAT however was superior with regard to sequestering non-T cell populations out of the CSF, including B cells, natural killer cells and inflammatory monocytes, suggesting that disease exacerbation in the context of switching might be driven by non-T cell populations. Finally, correlation of our immunological data with signs of disease exacerbation in this small cohort suggested that both (i) CD49d expression levels under NAT at the time of treatment cessation and (ii) swiftness of FTY-mediated effects on immune cell subsets in the PB together may predict stability during switching later on.

## Introduction

In multiple sclerosis (MS), immune cell trafficking plays a decisive role, and T-cell transmigration across the blood–brain-barrier (BBB) is tightly controlled (1–3). Two of the currently approved MS drugs, natalizumab (NAT) and fingolimod (FTY), act *via* selective interference with immune cell trafficking: NAT targets the α4-chain (CD49d) of the α4β1 integrin expressed on immune cells, therefore, directly interfering with adhesion to endothelial cell layers including the BBB (4), leaving peripheral immune cell subset composition mainly unaltered (5). FTY is a functional antagonist of the sphingosine-1-phosphate (S1P) pathway involved in mobilization of lymphocytes out of the secondary lymphatic organs and restrains CCR7 expressing lymphocytes from egressing into the peripheral blood (PB), which often results in peripheral lymphopenia (6).

In the context of MS, it is believed that both trafficking agents do not modify disease activity *per se*, instead both drugs “symptomatically” interfere with immune cell migration into the central nervous system (CNS), therefore, sheltering it from potential autoreactive lymphocytes. In consequence, upon treatment cessation, disease activity is rapidly reinstalled as immune cells restore their ability to migrate into their target organ and resume eliciting local inflammation, sometimes resulting in higher disease activity as compared to pretreatment levels (7–9).

One intriguing explanation underlying this highly relevant phenomenon of disease rebound might be an altered immunoregulatory network function due to long-term interference with the balance of effector and regulatory cell populations (10, 11). However, the consequences of therapeutic targeting of immune cell trafficking with regard to integrity and restoration of immune-tolerance networks have not yet been addressed.

## Materials and Methods

### Standard Protocol Approvals, Registrations, and Patient Consents

This 32-week, open, monocentric, exploratory, single-arm trial was conducted at the Department of Neurology, University of Münster as a follow-up study to the original ToFingo study to elucidate the immunological changes during switching in more detail (ClinicalTrials.gov: NCT01499667) (12). All patients met the eligibility criteria of diagnosis with relapsing–remitting multiple sclerosis (RRMS) according to the 2010 McDonald criteria (13). The full list of inclusion and exclusion criteria is outlined in Table S1 in Supplementary Material.

This follow-up study, ToFingo-successor, is registered at Clinicaltrials.gov (NCT02325440). Protocol and amendments were approved by the local ethics committee and the German competent authority (Federal Institute for Drugs and Medical Devices) in accordance with the Declaration of Helsinki. All participants provided written informed consent prior to entering the study.

### Patient Samples

For assessment of immunological parameters *via* flow cytometry, EDTA blood sampling was performed at each visit, PBMCs were isolated as described before (14) by density gradient centrifugation with Lymphoprep™ (STEMCELL technologies, Cologne, Germany) and cryopreserved in liquid nitrogen using serum-free cryopreservation medium (CTL-Cryo™ ABC Media Kit, Immunospot, Bonn, Germany) in concentrations of 1 × 10^7^ cells/ml. As controls, PBMCs of age- and sex-matched healthy donors (no previous history of neurologic or immune-mediated diseases) and treatment-naive RRMS patients were included in the analysis.

Additionally, to evaluate drug-induced changes of immune cell subset compositions in cerebrospinal fluid (CSF) under long-term NAT therapy compared to 6 months of FTY treatment, CSF specimen were obtained at baseline and at the end of the study course and immediately analyzed by flow cytometry in a subgroup of patients (*n* = 12 for NAT and *n* = 9 for FTY).

### Processing of PBMCs

Cryopreserved PBMCs were thawed in a 37°C water bath, resuspended in prewarmed cell culture medium RPMI + 10% FCS (Gibco™ life technologies, Carlsbad, CA, USA), and washed twice to remove the freezing medium. Subsequently, cells were used for flow cytometry or functional assays, respectively.

### Flow Cytometry and Monoclonal Antibodies

For surface-molecule staining, cells were washed with staining buffer [PBS (Sigma-Aldrich, Darmstadt, Germany), 0.1% FCS, 200 mM EDTA] and subsequently stained with fluorochrome-conjugated monoclonal antibodies. After washing once with staining buffer, cells were analyzed with a Navios flow cytometer (Beckman Coulter, Brea, CA, USA). For intracellular staining, cells were washed with Perm/Wash™ buffer (eBiosciences, Waltham, MA, USA), fixed for 20 min at 4°C with Cytofix/Cytoperm™ (eBiosciences, Waltham, MA, USA) and stained in Perm/Wash™ buffer for 30 min at 4°C. After repeated washing with Perm/Wash™ buffer and staining buffer, cells were analyzed. A full list of monoclonal antibody combinations for identification of cell populations is provided in Table S2 in Supplementary Material. Ensuing evaluation of flow cytometric data was performed with Kaluza software 1.5a (Beckman Coulter, Brea, CA, USA).

### Human Brain Microvascular Endothelial Cell (HBMEC) Culture and Cell Migration Assay

To assess the transmigratory capacity of immune cells across the BBB, an established cell migration assay was performed as described in detail before (14).

Shortly, on a monolayer of HBMECs (PELOBiotech GmbH, Planegg, Germany), thawed PBMCs (5 × 10^5^ cells per transwell) were added to the HBMEC monolayer in the upper compartment (3 × 10^4^ HBMECs per transwell) and the lower compartment was filled with transmigration medium (RPMI + 2% B27, Gibco™ life technologies, Carlsbad, CA, USA). After incubation for 6 h at 37°C flow count fluorospheres (Beckmann Coulter) were added for quantification of the migrated cells in the lower compartment, followed by cell surface staining and flow cytometry analysis. A well containing 5 × 10^5^ thawed PBMCs in 600 µl of RPMI + 2% B27 served as an *in vitro* control. The percentage of migrated cells for each cell type was calculated after the following equation:

$$\text{migrated cells (\%) = }\frac{\begin{array}{l} \text{absolute number of cells in }\\ \text{ lower compartment * }100 \end{array}}{\text{absolute number of cells in vitro}}$$

### Cytokine Secretion Assay

To evaluate the cytokine secretion of CD4^+^ T cells, thawed PBMCs were seeded at 5 × 10^5^ cells per 100 µl X-Vivo 15 cell culture medium (Lonza, Basel, Switzerland) in 96-well *U*-bottom plates (Greiner bio-one, Kremsmünster, Austria) and rested overnight at 37°C/5% CO_2_. Afterward, cells were either stimulated with Leukocyte Activation Cocktail (Phorbol 12-myristate 13-acetate, ionomycin, and Brefeldin A; BD Pharmingen™, CA, USA) X-Vivo 15 + 10 μl/ml or remained unstimulated for 6 h at 37°C/5% CO_2_. Finally, cells were washed and stained to analyze secretion of TNFα, IL-17A, GM-CSF, and IFNγ of CD3^+^CD4^+^ T cells by flow cytometry (Gallios, Beckman Coulter).

### MRI Acquisition

To observe disease activity during the trial period, MR imaging was performed at baseline, after the washout (WO) phase and at weeks 4, 8, 12, 20, and 24 of FTY treatment.

MRI acquisitions were performed with a 3T MRI scanner (TIM Trio, Siemens AG, Erlangen, Germany) and a 12-channel (matrix) head coil (Siemens AG, Erlangen, Germany). Images of the following MRI sequences were obtained: a native isotropic 3D MPRAGE T1-weighted sequence; axial turbo spin-echo (TSE) FLAIR; sagittal TSE FLAIR; 3D MPRAGE T1-weighted after intravenous gadolinium-diethylene triamine penta-acetic acid (DTPA) 0.1 mmol/kg injection; axial PD/T2-weighted TSE. T1-weighted images before and after administration of contrast medium (0.1 mmol/kg gadolinium-DPTA) as well as T2-weighted (T2 and PD) images were assessed. MRI disease activity was defined as change in T2w lesion load, detection of gadolinium-enhancing (Gd+) lesions as well as changes in T1w and FLAIR lesions compared to the baseline MRI.

### Correlation of Clinical and Immunological Response

To assess potential correlation of peripheral immune signatures with occurrence of clinical and/or paraclinical disease activity, the study cohort was divided into three subgroups: “stable” (*n* = 6) “intermediate” (*n* = 5), and “exacerbated” (*n* = 4). Stable patients were defined as patients with no evidence of disease activity, absence of relapses, EDSS progression, and new Gd+ or T2w lesions throughout the study. Patients suffering from a relapse or exhibiting profound MRI activity (i.e., ≥5 new Gd+ lesions) were defined as “exacerbated.” All other patients showing mild MRI activity were grouped as “intermediate.”

### Visualization of High-Dimensional Single-Cell Data

To visualize 10-color flow cytometry data, we applied viSNE, an unbiased visualization tool implemented in *cyt*, written in MATLAB^®^. The Barnes-Hut Stochastic Neighbor Embedding (bh-SNE) algorithm maps single cell data points from high dimensional- to low dimensional space by minimizing the difference in pairwise similarities between points (15, 16). FCS files of study participants were randomly subsampled, merged, and subsequently mapped into a 2D bh-SNE plot. To reduce sample-to-sample acquisition variability, a normalization algorithm (17) was applied to the datasets prior to clustering. To promote divergence and avoid “crowding” of clusters, data point size was limited to 100,000 points in total. We applied Phenograph (18) to identify populations (affiliations are indicated by color-coding circles). Heatmap illustrates twofold change of reduced- (green) and increased (red) percental differences of populations comparing baseline (NAT) to study endpoint (FTY24). Spanning-tree progression analysis of density-normalized events (SPADE), a further cluster analysis, begins by density-dependent downsampling of the original data and applies hierarchical clustering to this subset of cells (19). After upsampling, the resulting groups of cell types (nodes) are visualized using a minimal spanning tree plot, in which nodes of close proximity also display phenotypical similarity. The color of the node was scaled to the ratio of change in cell frequency between baseline and study endpoint (FTY24). Putative cell populations were semiautomatically annotated around sets of nodes of comparable characteristic CD-marker expression using an edge removal algorithm (20). The target number of nodes was fixed to 100 for global or 50 for subset analysis by empirically evaluating results from multiple SPADE runs on the same dataset, based on the number of expected populations, their relative size, and the total number of cells in the dataset.

### Statistical Analysis

Statistical analysis was performed in collaboration with the Institute of Biostatistics and Clinical Research, University of Münster. To account for dependencies of repeated measurements, linear mixed models were used to analyze the course of different parameters from NAT baseline to FTY 24 weeks. To compare visits, a model including the variable visit as fixed effects as well as a random intercept for each patient was used. Based on these models, adjusted least square means and corresponding *t*-tests were used for comparisons. All inferential statistics are considered exploratory and, therefore, no adjustment for multiple testing was applied. Statistical analysis was performed by SAS version 9.4 (SAS Institute, Cary, NC, USA).

## Results

### Broad but Distinct Effects of the Trafficking Agents NAT and FTY on Peripheral Immune Signatures

**Figure 1:**
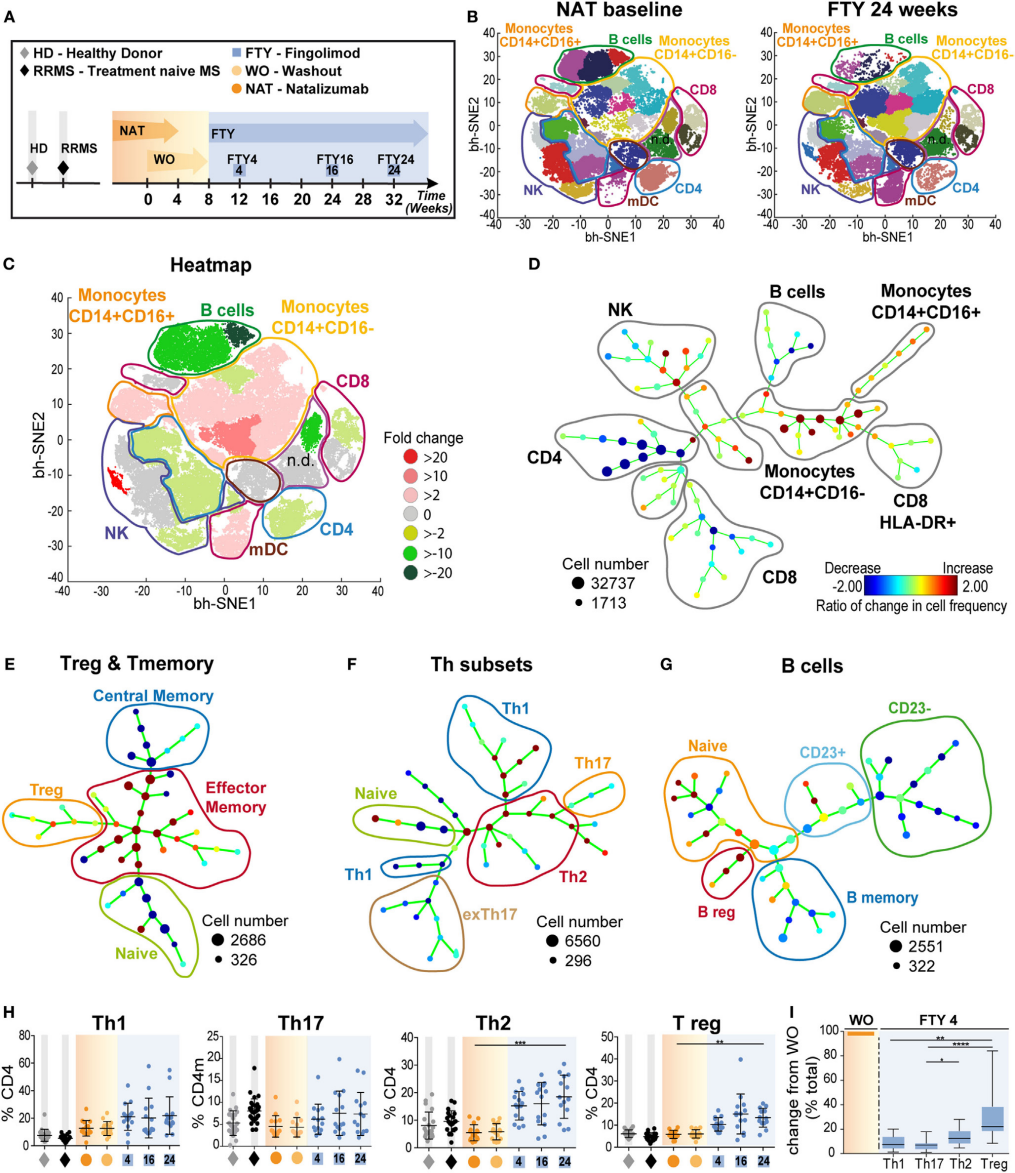
Overview of comprehensive changes of immune cell subset compositions and differential effects on pro- and anti-inflammatory T cells **(A)** Study setup; data (*n* = 15) were assessed at baseline under long-term natalizumab (NAT) treatment (orange), after 8 weeks of washout without treatment (WO; light orange), and at time points 4 weeks (FTY4; blue), 16 weeks (FTY16; blue), and 24 weeks (FTY24; blue) after onset of fingolimod treatment indicated by arrows. Healthy donors (HD; gray) and treatment-naive relapsing–remitting multiple sclerosis (RRMS) patients (RRMS; black) represent control groups. **(B)** Visualization of 10-color flow-cytometry data *via* viSNE application; merged data of study participants at indicated time points in single cell dot plots (NAT *n* = 13; FTY24 *n* = 13). Affiliations of populations are indicated by color-coding circles and labeling; n.d., not defined population. **(C)** Heatmap illustrates twofold change of reduced (green) and increased (red) percental differences of populations comparing baseline (NAT) to study endpoint (FTY24). **(D)** Spanning-tree progression analysis of density-normalized events (SPADE) diagram compares long-term NAT with FTY24 and nodes represent groups of cell types in which the color of the node is scaled to the ratio of change in cell frequency; close proximity of the nodes display phenotypical similarity. **(E–G)** SPADE analysis (*n* = 13) compares long-term NAT-treated patients to study endpoint (FTY24) and nodes represent ratio of the change of cell frequency of **(E)** regulatory T-cells, CD4 subpopulations, **(F)** T-helper subsets, and **(G)** of B cell subsets. **(H)** Relative proportions (in %) of flow cytometry data of T-helper subpopulations for indicated time points. **(I)** Change in absolute cell counts after treatment switch by comparison of FTY4 to the WO time point (in % total). Values represent mean ± SD. Statistical significance was evaluated by linear mixed model; Friedman test (one-way ANOVA). *P ≤ 0.05; **P ≤ 0.01; ***P ≤ 0.001; ****P ≤ 0.0001.

Here, we made use of a small, highly controlled clinical study cohort of 15 RRMS patients under long-term treatment with NAT, who were switched to FTY after an 8-week WO interval and followed up for another 6 months (“ToFingo successor study” Figure 1A; cohort description in Table 1; and Table S1 in Supplementary Material). We first performed a detailed, multiparameter immune cell profiling by flow cytometry followed by unbiased visualization of high-dimensional single cell flow cytometry data by viSNE and SPADE to get a comprehensive overview about immune cell subset composition and activation status in the periphery of MS patients under treatment with NAT versus FTY in comparison to healthy controls and treatment-naive RRMS patients. These unsupervised machine-learning tools exposed changes in peripheral cellular composition after treatment switch, revealing that switching from one trafficking drug to another essentially affected the proportions of more than 80% of all immune cell subpopulations investigated, as is evident from the viSNE maps before and after change of treatment (Figure 1B). To further illustrate the most relevant changes, we created a heatmap of the combined viSNE maps based on the fold-change differences of the Phenograph clusters before and after FTY treatment (Figure 1C). Additionally, we corroborated these findings by SPADE analysis in 10-dimensional space (Figure 1D). Here, the nodes were colored according to the change in cellular frequency after treatment switch (cf. Materials and Methods). Also in the SPADE tree plot, the majority of cluster frequencies changed upon FTY treatment, with the most prominent changes occurring within the monocyte, B cell, and T cell compartment. This suggests that despite the specific and distinct mode of action of both drugs, interference with immune cell trafficking results in a profound and complex alteration of the balance between numerous effector- versus regulatory immune cell populations as measured in PB.

**Table 1:** Summary of baseline demographics.

	ToFingo patients (*n* = 15)	Naive multiple sclerosis (MS) (peripheral blood) (*n* = 19)	Naive MS (cerebrospinal fluid) (*n* = 12)	Healthy donor (*n* = 21)
Sex F/M	11/4	12/7	8/4	14/7
Age, years, mean ± SD (range)	38.8 ± 9.2 (25–53)	44.47 ± 14.59 (21–68)	44.8 ± 17.9 (23–78)	44.00 ± 11.05 (22–56)
MS duration since first symptoms, years, mean ± SD	11.5 ± 6.7	7 ± 6.7	6.7 ± 8.4	
MS duration since confirmed diagnosis, years, mean ± SD	8.9 ± 6.9	6.3 ± 6.3	4.2 ± 7.3 (*P* = *)	
EDSS baseline, mean ± SD	2.2 ± 1.1	2.5 ± 1.88	1.9 ± 1.1	
EDSS EOS, mean ± SD	2.4 ± 1.5			
**Any previous MS treatment prior to NAT, *n* (%)**			
Glatiramer acetate	9 (60.0)			
Interferon-β	11 (73.0)			
Azathioprine	1 (6.0)			
Positive JC virus status, *n* (%)	15 (100.0)			
Existing T2w lesions at baseline, *n*, mean ± SD (range)	30 ± 9.1 (15–43)			

EDSS, Expanded Disability Status Scale; EOS, end of study; statistical analysis by Mann–Whitney U-test; *P ≤ 0.05.

To further characterize the sequential changes in immune cell subset composition, we first analyzed cell subsets in detail *via* SPADE and, next, we corroborated these results by analysis of cell frequencies and absolute numbers *via* conventional flow cytometry. After long-term NAT treatment and after FTY initiation, we confirm several known drug-related changes (21); i.e., relative increase of innate populations and preferential retaining of CCR7^+^ expressing naive and central memory (cm) T cells (Figure 1E; Figure S1 in Supplementary Material). Intriguingly, for T- helper cells (Th)-subpopulations, our unbiased analysis *via* SPADE revealed a relative rise in both T- helper cells 2 (Th2) and regulatory T cell (Tregs) proportions under FTY compared to NAT illustrated by changed nodes (Figures 1E,F), whereas for Th1 and Th17 cell proportions, no major changes could be observed. Accordingly, we observed a preferential reduction in memory B cells under FTY treatment, whereas there was a striking increase in regulatory B cell populations during FTY treatment, as described before (14, 22) (Figure 1G; Figures S2A,B in Supplementary Material). Further quantification of the relative changes in Th-subpopulations demonstrated a significant increase of Th2 and Treg populations in the periphery (Figure 1H). In line with the mechanism of action of FTY, absolute counts of all Th-subpopulations decreased (Figure S2C in Supplementary Material); however, reductions of Th2 and Treg were less pronounced compared to those of Th1 and Th17 cells, respectively (Figure 1I).

### CSF Analysis Reveals Differential Efficacy of NAT and FTY on Non-T Cell Populations

**Figure 2:**
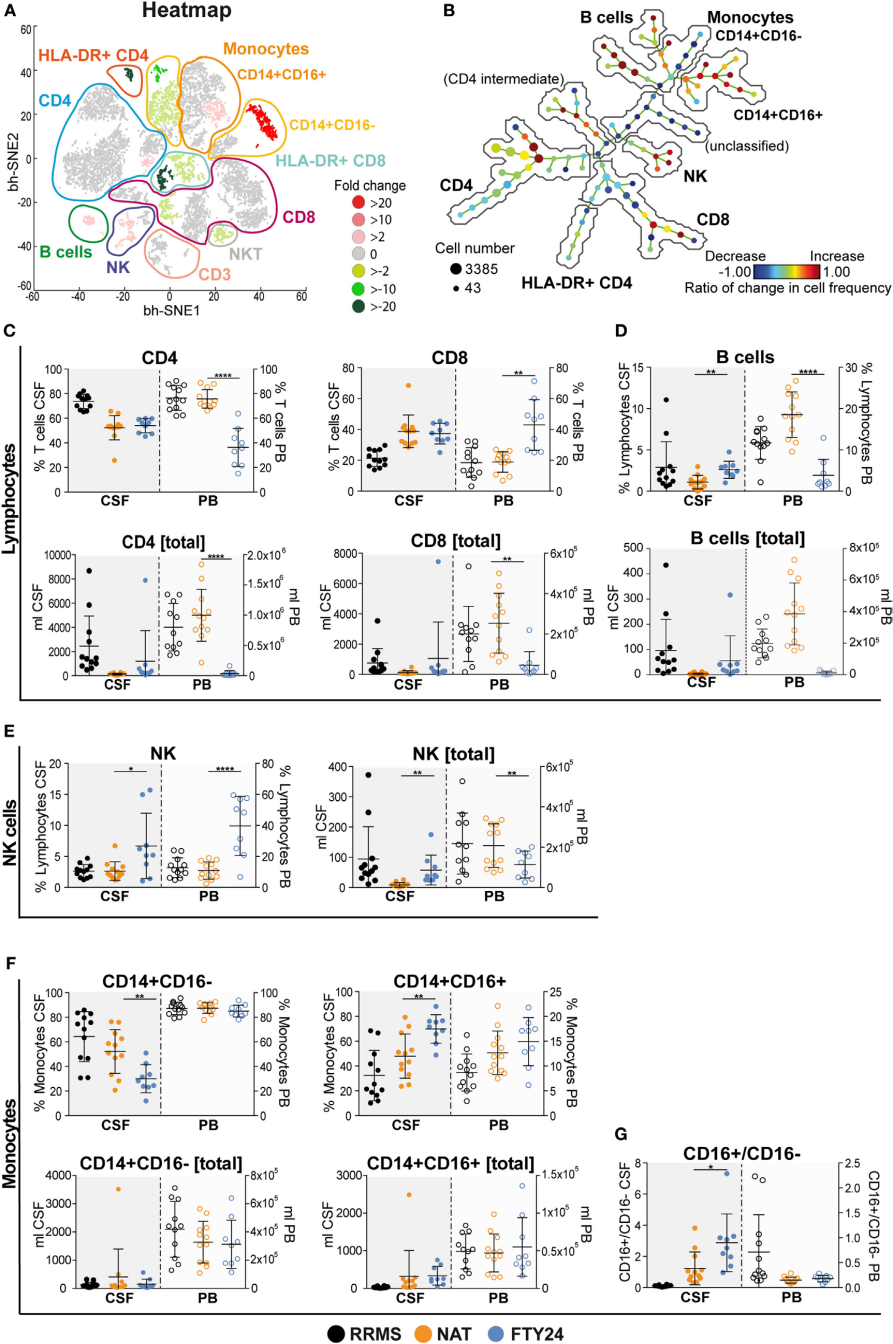
Natalizumab (NAT) inhibits non-T cell invasion into the cerebrospinal fluid (CSF) more efficiently than fingolimod (FTY). Assessment of flow cytometry data attained from fresh CSF and corresponding peripheral blood (PB) samples, at baseline under long-term NAT treatment (NAT; *n* = 12) and 24 weeks after onset of FTY treatment (FTY24; *n* = 9). **(A)** Non-linear neighbor embedding by viSNE application; heatmap represents twofold change increase (red) or decrease (green) of populations comparing baseline (NAT; *n* = 9) to study endpoint (FTY24; *n* = 9) of study participants. Affiliations of populations are indicated by color-coding circles and labeling; n.d., not-defined population. **(B)** Spanning-tree progression analysis of density-normalized events diagram compares CSF samples of long-term NAT with FTY24 and nodes represent groups of cell types in which the color of the node is scaled to the ratio of change in cell frequency; close proximity of the nodes display phenotypical similarity. **(C–G)** Relative proportions (in %) and total counts (per milliliters) of **(C)** CD4 and CD8 T cells, **(D)** B cells, **(E)** natural killer cells, **(F)** CD14^+^CD16^−^ and CD14^+^CD16^+^ monocytes, and **(G)** CD16^+^/CD16^−^ ratio were assessed in CSF (closed dots) and PB (open dots) comparing FTY (blue) to NAT (orange) treatment; control group naive multiple sclerosis patients (relapsing–remitting multiple sclerosis; black). Values represent mean ± SD. Statistical significance was evaluated by linear mixed model. *P ≤ 0.05; **P ≤ 0.01; ****P ≤ 0.0001.

Peripheral blood can only partially reflect drug-related changes for immune cell trafficking with regard to the target organ. Therefore, we next characterized immune cell subset frequencies as well as absolute cell counts within the CSF in our cohort both under long-term NAT treatment and 6 months after FTY treatment initiation (*n* = 12 under NAT and *n* = 9 out of 12 under FTY), thus allowing for a direct comparison within the same individuals. viSNE analysis revealed numerous changes in different immune cell subpopulations illustrated by heatmap (Figure 2A). The unsupervised organization of cell types *via* the SPADE algorithm also indicated a substantial change to baseline, as reflected by the ratios of change in cell frequencies in numerous nodes of the SPADE tree plot (Figure 2B). Both our unbiased approaches revealed that changes in immune cell frequencies between NAT and FTY are most prominent in non-T cell and innate immune cell populations such as B cells, monocytes, and natural killer (NK) cells (Figures 2A,B).

Further conventional analysis of our flow cytometry data suggested a slight increase in total leukocyte- and lymphocyte numbers (Figure S3A in Supplementary Material) and CD4 and CD8 T cell counts (Figure 2C) within the CSF under FTY in comparison to NAT, albeit this did not reach significance. Furthermore, total leukocyte and lymphocyte counts under FTY were still lower than those of untreated RRMS patients (Figure S3A in Supplementary Material). Interestingly, both CD4 and CD8 T cell frequencies as well as absolute counts remained stable in the CSF in all patients during switching (Figure 2C; Figure S3B in Supplementary Material). In contrast, the most striking differences in CSF signatures were seen on non-T cell subsets: B cell proportions in the CSF were significantly increased under FTY versus NAT treatment (Figure 2D), which was also true for NK cell proportions (Figure 2E). Also a slight increase in plasma cell proportions could be observed (Figure S3C in Supplementary Material). Furthermore, there was a shift in monocyte subpopulations in the CSF under FTY treatment, with a decrease in CD16^−^ and a reciprocal increase in CD16^+^ monocyte subsets, hence reflecting a preferential enrichment of proinflammatory monocytes in the CSF under FTY treatment (Figures 2F,G; Figure S3D in Supplementary Material). This is supported by analysis of absolute counts as we observed a significant increase in absolute numbers in NK cells and proinflammatory monocytes and a robust tendency in B cell counts (Figures 2D–G).

In summary, our detailed longitudinal analysis of immune cell subsets and signatures within the CSF revealed that both NAT and FTY were equally efficient in restricting T cell populations from entering the CSF; however, in the presence of NAT, non-T-cell populations including B cells, NK cells, and inflammatory monocytes were significantly less present in the CSF compared to FTY.

### Impact of Immune Trafficking Agents on Markers of T Cell Function

**Figure 3:**
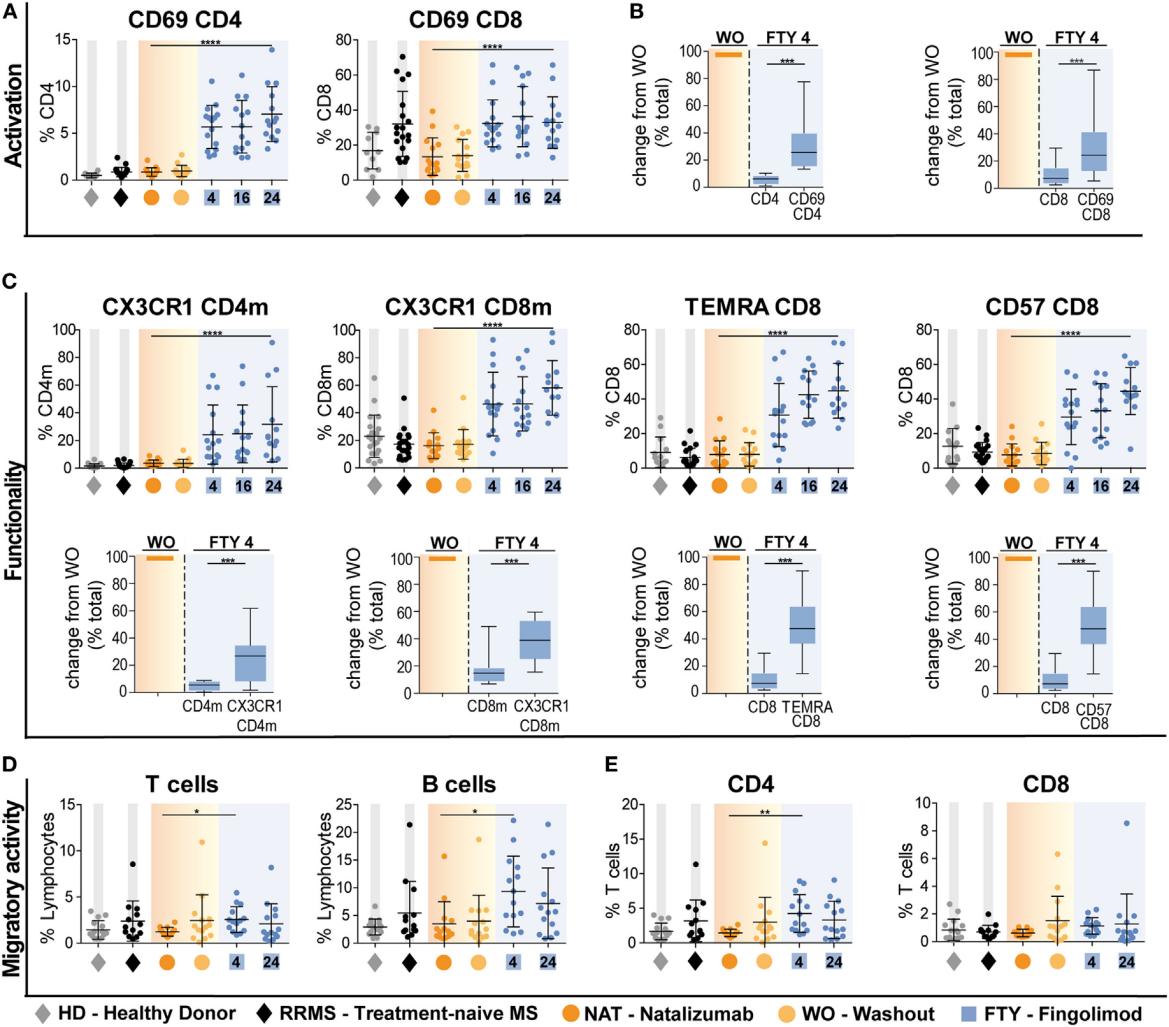
Preferential enrichment of terminally differentiated and functionally highly competent effector T cell subsets under fingolimod (FTY) treatment. All data (*n* = 15) were assessed at baseline under long-term natalizumab treatment (NAT), after 8 weeks of washout without treatment (WO) and at time points 4 weeks (FTY4), 16 weeks (FTY16), and 24 weeks (FTY24) after onset of FTY treatment. Healthy donors (HD) and treatment-naive relapsing–remitting multiple sclerosis (RRMS) patients (RRMS) represent control groups. Scatter plots illustrate relative frequency changes (%) of **(A)** activation marker CD69^+^ CD4 and CD69^+^ CD8 T cells. **(B)** Change in absolute cell counts after treatment switch by comparison of FTY4 to the WO time point (in % total) of CD69^+^ CD4 and CD69^+^ CD8 counts to total CD4 and CD8 counts. **(C)** Relative proportions (in %) of flow cytometry data of functionality markers CX3CR1^+^ CD4 memory (CD4m) and CX3CR1^+^ CD8 memory (CD8m), TEMRA CD8 and CD57^+^ CD8 T cells comparing FTY to NAT treatment at indicated time points. Absolute change after treatment switch comparing CX3CR1^+^ CD4m and CX3CR1^+^ CD8m total counts to total CD4 and CD8 memory counts, as well as TEMRA^+^ CD8 and CD57^+^ CD8 to absolute counts of CD8 at time points WO to FTY4 (in % total). **(D,E)** Transmigration assay was used to assess migratory capacity in percent, through human brain microvascular endothelial cell monolayer for 6 h in an *in vitro* model determined for **(C)** T cells and B cells, **(D)** CD4 and CD8 T cells comparing FTY and NAT treated patients to HD and RRMS controls. Values represent mean ± SD. Statistical significance was evaluated by linear mixed model, Kruskal–Wallis test, and Wilcoxon matched-pairs signed rank test. *P ≤ 0.05; **P ≤ 0.01; ***P ≤ 0.001; ****P ≤ 0.0001.

In the next set of experiments, we addressed whether interference with immune cell trafficking might alter immune cell function. As a first approach, we compared expression of activation markers and molecules associated with terminal differentiation and functionality of T cells. Interestingly, there was a striking increase in the proportion of CD4 and CD8 T cells expressing the early activation marker CD69 in FTY-treated patients (Figure 3A). This is supported by analysis of absolute cell counts, as numbers of activated CD4 and CD8 cells were less reduced as compared to those of total CD4 and CD8 cells (Figure 3B, Figure S4A in Supplementary Material). In this line, also proportions of T cells expressing markers, which identify highly functional terminally differentiated T cells such as CX3CR1, CD57, and CCR7^−^CD45RA^+^ (TEMRA markers) were significantly increased under FTY but not NAT treatment and changes of absolute count revealed that highly functional T cells were less affected by FTY as compared to total cell count (Figure 3C, Figure S4B in Supplementary Material). Although this might suggest that FTY treatment may directly or indirectly enhance T cell activation, we propose that it rather reflects the decreased susceptibility of highly differentiated cells toward FTY/S1P-mediated trapping.

This is further supported by functional data comparing the migratory- and cytokine-producing capacity of T cell subsets under NAT versus FTY treatment: employing an established *in vitro* model of the BBB (14), T cells from FTY-treated patients did not exhibit an altered migratory capacity when compared to those of treatment-naive RRMS patients and this was true for both CD4 and CD8 T cell subpopulations (Figures 3D,E). In line with the mode of action of NAT, T cells and B cells from NAT-treated patients exhibited a significantly lower migratory activity compared to those from treatment-naive patients as well as those under FTY (Figures 3D,E).

Of note, proinflammatory cytokine production by stimulated CD4 T cells, i.e., IFNγ, TNFα, IL-17A, and GM-CSF, was not altered during switching from NAT and FTY, indicating that cytokine-producing capacity *per se* is not affected by both trafficking agents (Figure S4C in Supplementary Material).

### Correlation of Immune Signatures With Signs of Disease Exacerbation

**Figure 4:**
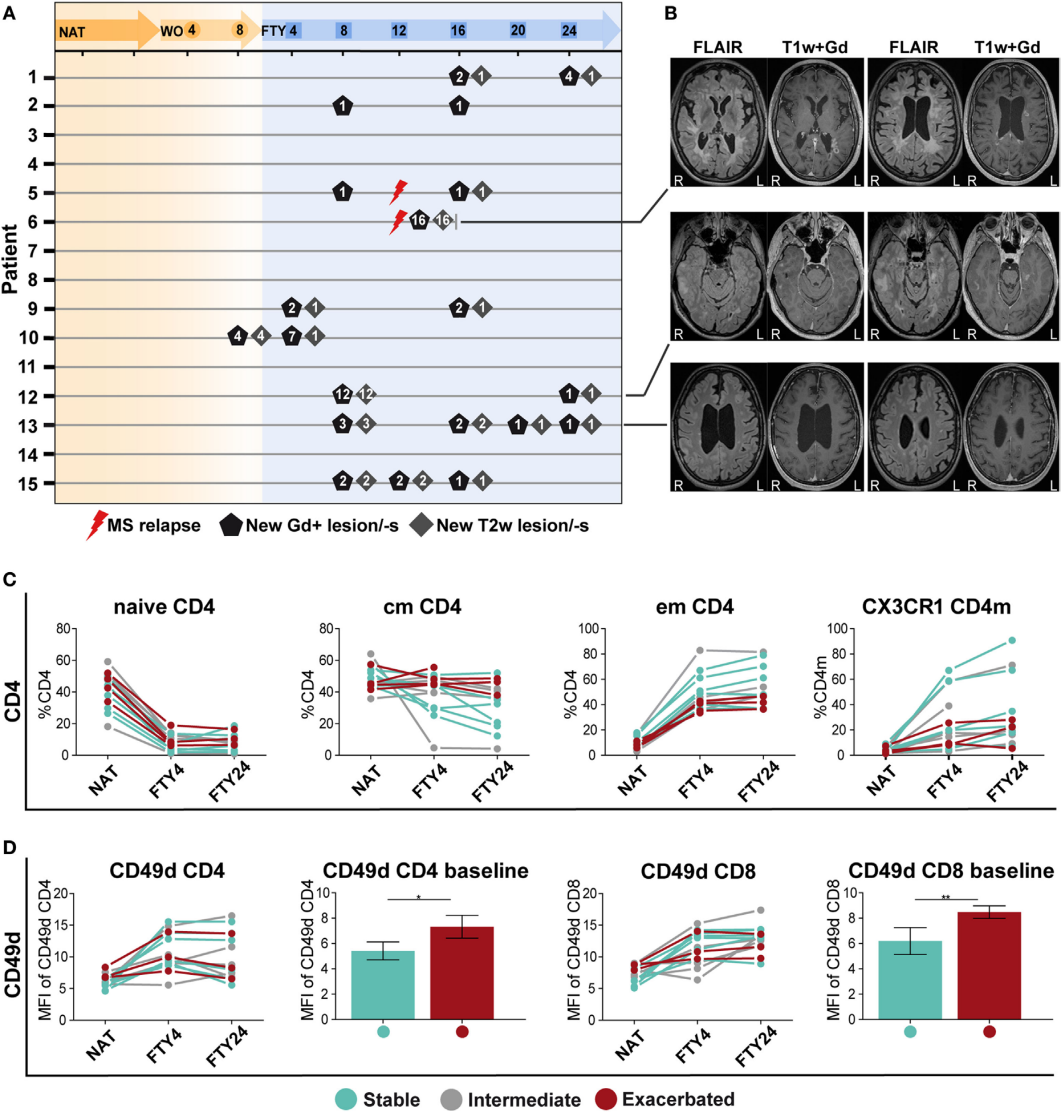
Patients with a stable disease course exhibit significantly reduced CD49d expression levels under long-term natalizumab (NAT) treatment. **(A)** MR imaging was performed at baseline under long-term NAT treatment (NAT), after 8 weeks of washout without treatment (WO) and at weeks 4, 8, 12, 20, and 24 after onset of fingolimod (FTY) treatment. Numbers of new gadolinium-enhancing (Gd+)-lesion/-s (black pentagon), T2w-lesion/-s (gray square), and clinical multiple sclerosis relapses (red flash) are presented per patient and time point. **(B)** Examples of the MRI sequences FLAIR and T1-weighted after intravenous gadolinium-DTPA injection (T1w + Gd) of patients no. 6, 11, and 13 who were defined as “exacerbated” are displayed (R = right; L = left). To assess correlation of peripheral immune signatures with clinical response, the study cohort was divided into subgroups: stable (blue; *n* = 6), intermediate (gray; *n* = 5), and exacerbated (red; *n* = 4 time point NAT and FTY4; *n* = 3 time point FTY24) estimated by clinical criteria. **(C)** Comparison of population frequency (%) between baseline long-term NAT treatment (NAT), at time points 4 weeks (FTY4) and 24 weeks (FTY24) after onset of FTY treatment, in CD4 T cell subsets, and CX3CR1^+^ CD4 memory (CD4m) T cells are shown. **(D)** MFI of CD49d expression on CD4 and CD8 T cells at indicated time points illustrated in scatter plots and direct comparison of CD49d CD4 and CD8 between stable and exacerbated patients at baseline shown in bar graphs. Values represent mean ± SD. Statistical significance was evaluated by Wilcoxon matched-pairs signed rank test. *P ≤ 0.05; **P ≤ 0.01.

Finally, we analyzed peripheral and CSF immune signatures in correlation to treatment response profiles as reflected by clinical and radiological signs of disease activity. To this end, we grouped patients who remained completely free of any clinical or radiological disease activity during switching from NAT to FTY (*n* = 6), and those who exhibited disease exacerbation (*n* = 4) as defined by either presence of a confirmed clinical relapse and/or detection of substantial new MRI disease activity as described in detail in Section “Materials and Methods” and depicted in Figures 4A,B.

When comparing proportional and absolute changes in CD4 and CD8 T cell subset composition during switching between these predefined subgroups, we consistently observed that stable patients exhibited stronger FTY-mediated immunological alterations as those suffering from disease exacerbation (Figure 4C). Changes were most prominent in cmCD4, emCD4, CX3CR1 CD4m, and CX3CR1 CD8m T cells potentially reflecting swift immunological response to FTY treatment (Figure 4C; Figures S5A,B in Supplementary Material), although no statistical significance can be attributed due to the low patient numbers. Also within B cell subsets, such a correlation was suggested for regulatory B cells but not for other B cell populations (Figure S5C in Supplementary Material), in analogy to previous reports (14, 22).

Finally, the extent of NAT-elicited immunological changes might affect the clinical response during switching later on. We, therefore, assessed the expression levels of CD49d on CD4 and CD8 T cell subsets over time, as CD49d expression is downmodulated during NAT treatment (23, 24). Interestingly, we observed that those patients, who remained stable later on, exhibited significantly lower CD49d expression levels on CD4 and CD8 T cells at baseline as compared to those suffering from disease exacerbation (Figure 4D). Together, these data indicate that both the individual efficacy of NAT at the time of treatment cessation as reflected by CD49d expression levels on T cells and the extent and swiftness of FTY-mediated effects on immune cell subsets in the PB together may predict whether patients remain stable during switching from NAT to FTY.

## Discussion

This study provides direct insights in comparing the differential immune cell signatures of two drugs interfering with leukocyte trafficking in MS within a controlled monocentric investigator-initiated clinical trial. Comprehensive unbiased analysis of our flow-cytometry data *via* viSNE and SPADE of longitudinal drug-related changes both in the periphery and the CSF allowed for the first time a direct comparison of the differential effects of both drugs. While both drugs had a considerable impact on multiple peripheral immune subsets, NAT was more efficient in restricting non-T cell invasion (B cells, NK cells, and monocytes) into the CSF. Our results strongly suggest that drug-induced changes in immune cell trafficking have a considerable and so far underestimated impact on immunoregulatory network function.

In line with previous publications concerning the mechanism of action of NAT and FTY, we confirmed that the 8-week washout period of NAT did not substantially alter peripheral lymphocyte quantities (25), while FTY treatment led to a profound decrease in lymphocyte counts, primarily by retaining CCR7^+^ naive and cm CD4 and CD8 T cells as well as, with a slight delay, B cells within the lymphatic tissue (Figures 1E–G; Figures S1 and S2A,B in Supplementary Material). When assessing the functional properties of CD4 T cells from long-term NAT- and FTY-treated patients, no changes in the production of proinflammatory cytokines such as IFNγ, TNFα, and IL-17A and GM-CSF were observed (Figure S4C in Supplementary Material), indicating that the functional capacities of T cells at least with regard to cytokine production were not affected by both trafficking agents. This is in accordance with a previous study that could not reveal significant alterations of cytokine secretion on *in vitro* NAT-treated CD4 T cells (10). Moreover, changes of functional properties on an individual cell level during FTY therapy have not been described in literature. However, we observed a preferential enrichment of terminally differentiated and functionally highly competent effector CD4 and CD8 T cells as illustrated by increased proportions of CD69, CX3CR1, CD57, and CCR7^−^CD45RA^+^ (TEMRA) expressing cells in the periphery of FTY-treated patients (Figures 3A–C), suggesting that the trapping indirectly favors the presence of highly competent cells in the peripheral circulation. This indicates that despite drug-induced peripheral lymphopenia with potentially reduced quantitative immune responses as, for example, observed during vaccinations (26), such preferential enrichment of highly competent lymphocyte subsets may at least partially sustain immune competence despite absolute reductions in peripheral lymphocyte numbers (21). Analysis of the migratory capacity of peripheral immune cells employing an *in vitro* model of the BBB (14) revealed that FTY patients exhibited a higher migratory capacity as compared to NAT-treated patients, either due to the persisting binding of NAT to CD49d-expressing immune cells or due to enrichment in proportion of T memory subpopulations with an enhanced migratory potential in line with the mode of action of FTY (Figures 3D,E). In our study, we had the unique opportunity to directly compare the effects of NAT versus FTY on CSF immune cell subset composition within the same individuals, which has not been addressed before, at least to our knowledge. This intraindividual comparison allowed several conclusions: first, both drugs displayed very similar effects on CD4 and CD8 T-cell counts and proportions within the CSF, hence suggesting that both display a similar efficacy with regard to inhibition of T cell trafficking into the CNS (Figure 2C). Second, we observed that NAT was superior to FTY with regard to control non-T cell invasion into the CNS, as B cell, NK cell and inflammatory monocyte proportions were significantly increased under FTY treatment (Figures 2D–G). This suggests that the postulated superior efficacy of NAT at least in some individuals as described by studies on observational cohorts (27, 28) might be rather due to a superior control of non-T-cell invasion than control of T-cell transmigration itself, thus further underlining the increasingly appreciated role of non-T cells in MS pathophysiology (29–31).

In light of the well-known heterogeneity in clinical response during switching from NAT to FTY, we searched for immunological signatures potentially associated with disease stability during switching by comparing immune profiles of stable versus exacerbated patients. Due to the limited sample size of these subgroups, a statistical analysis was not possible; however, disease stability during switching seemed to be associated with an early and pronounced FTY effect illustrated by reduced cmCD4 T cells and increased emCD4, CX3CR1 CD4m, and CD8m T cells already 4 weeks after treatment onset (Figure 4C; Figures S5A,B in Supplementary Material). Furthermore, since CD49d expression has received attention in relation to treatment response to NAT in previous reports (23, 24), we also compared the CD49d expression kinetics in stable versus exacerbated patients (Figure 4D). Interestingly, no difference in the kinetics of CD49d recovery upon NAT treatment cessation could be observed; however, those with subsequently stable disease course exhibited significantly reduced CD49d expression levels under long-term NAT treatment, indicating that the extent of NAT-induced reduction in CD49d levels but not the kinetics of recovery might predict stable disease course during switching to another treatment.

Our multidimensional analysis of flow cytometry data revealed that switching from NAT to FTY had a rather extensive impact on peripheral immune signatures (Figures 1B–D). This suggests that targeting of immune cell trafficking despite its apparently selective effects on distinct subsets finally has a relevant impact on the balance between numerous proinflammatory and regulatory immune cell subsets. This implicates that pharmacological targeting of immune cell trafficking may indirectly influence immunoregulatory network function, which has not been recognized before to such an extent. Several studies already pointed in this direction: for example, for FTY, a proportional increase in Tregs (11, 32), Bregs, and transitional B cells and a concomitant decline in proportions of memory B cells (14, 22, 33, 34) have been reported. We now contribute to these findings, as we not only observed significantly elevated proportions of Tregs but also a relative increase in anti-inflammatory Th2 cells under FTY treatment as compared to NAT, whereas proinflammatory Th1 and Th17 cell proportions remained relatively unaffected (Figures 1H–I) shifting the balance toward anti-inflammatory subsets. In the future, more detailed investigation into Th-subpopulations is warranted to provide further new insights into the understanding of the restoration of immunoregulatory network function.

From a more general perspective, the concept of drug-induced rebalancing of disturbed immunoregulatory network functions in MS patients has already been proposed and investigated in the context of cell-depleting strategies such as alemtuzumab (35) and rituximab (36), which act *via* selective ablation of certain immune cell subsets followed by a distinct repopulation pattern of regulatory and effector subpopulations (37, 38). Moreover, the recent example of daclizumab (39), which not only acts through direct blockade of activated CD25 T cells but also restricting myeloid dendritic cells from activating T cells and expansion of regulatory NK cell subsets due to increased levels of circulating IL-2 reveals that restoration of network balance affects both adaptive and innate immune cell compartments further elucidating a critical mutual dependency (40, 41).

Our analysis of immunoregulatory network functions allows the conclusion that drugs originally referred to as genuine “trafficking agents” may ultimately interfere with immune-tolerance networks, hence shedding new light on the diverse mechanisms of action of these “well-understood” drugs. In other words, these trafficking agents might not primarily act *via* targeted sequestration of an individual immune cell subset but rather by re-shaping the balance between different immune cell subsets, therefore, restoring immunoregulatory network function. This concept may have broad implications also with regard to clinical applications, as the unidimensional interpretation of trafficking blockade did not satisfactorily explain the well-known interindividual differences in response rates. Of note, such differential response rates are more common in the context of FTY (42), which in line with our data may suggest that here the influence on immunoregulatory network function, as opposed to direct interference with effector cell trafficking, might be more important as compared to NAT (43).

Finally, it should be noted that this exploratory study was performed in a small number of patients and inferential statistics are considered exploratory and, therefore, no adjustment for multiple testing was applied. As a consequence of analysis of multiple parameters, this approach is prone to false positive results. We tried to address this limitation by (i) use of unbiased multiparameter analysis tools such as viSNE and SPADE, which avoid singular examination of isolated parameters, and by (ii) combined interpretation of several findings corroborating each other, as for example, the effector molecule signatures caused by FTY (Figure 3). However, these results need to be confirmed in a larger independent cohort. A better understanding of the patterns of disturbed immunoregulatory network functions in MS patients will in the future pave the path for a more individualized treatment choice.

## Ethics Statement

This study was carried out in accordance with the recommendations of ICH Harmonized Tripartite Guidelines for Good Clinical Practice with applicable local regulations (including European Directive 2001/20/EC) and with the ethical principles laid down in the Declaration of Helsinki. The protocol was approved by the ethic commission of the “Aerztekammer Westfalen-Lippe und der Westfaelischen Wilhelms-Universitaet Muenster” (2014-060-J-A; 2010-262-J-S). All subjects gave written informed consent according to the study protocol.

## Author Contributions

Concept and study design: LKlotz, HW, NS, TS-H, CG, and SM; data acquisition and analysis: LL, CJ, AS-M, SK, LKirstein, UH, TW, and ME; data interpretation: LKlotz, HW, LL, and CJ; drafting of the manuscript and figures: LKlotz, LL, and CJ; editing and revision of the manuscript: all authors.

## Conflict of Interest Statement

LL, AS-M, SK, LKirsten, UH, TW, and ME have nothing to disclose. LKlotz: received compensation for serving on Scientific Advisory Boards for Genzyme and Novartis; received speaker honoraria and travel support from Novartis, Merck Sorono, Biogen, and Genzyme; and receives research support from Novartis and Biogen. HW: received honoraria for acting as a member of Scientific Advisory Boards and steering committees for Biogen, Evgen, MedDay Pharmaceuticals, Merck Serono, Novartis, Roche Pharma AG, Sanofi-Genzyme, as well as speaker honoraria and travel support from Alexion, Biogen, Cognomed, F. Hoffmann-La Roche Ltd., Gemeinnützige Hertie-Stiftung, Merck Serono, Novartis, Roche Pharma AG, Peervoice, Sanofi-Genzyme, Swiss Multiple Sclerosis Society, TEVA, and WebMD Global; he acted as a paid consultant for Abbvie, Actelion, Biogen, IGES, Novartis, Roche Pharma AG, Sanofi-Genzyme, and GlaxoSmithKline GmbH; received research support from the German Ministry for Education and Research (BMBF), Deutsche Forschungsgesellschaft (DFG), Else Kröner Fresenius Foundation, Fresenius Foundation, Hertie Foundation, NRW Ministry of Education and Research, Novartis, Interdisciplinary Center for Clinical Studies (IZKF) Muenster and RE Children’s Foundation, Biogen, GlaxoSmithKline GmbH, Roche Pharma AG, Sanofi-Genzyme. SM: received honoraria for lecturing, travel expenses for attending meetings and financial research support from Almirall, Bayer Healthcare, Biogen, Chugai Pharma, Diamed, Genzyme, MedDay Pharmaceuticals, Merck Serono, Novartis, Novo Nordisk, ONO Pharma, QuintilesIMS, Roche Pharma AG, Sanofi-Aventis, and Teva. CJ: received travel support from Novartis. NS: received travel support from Sanofi-Genzyme and Novartis. TS-H: received travel support from Biogen, Bayer Healthcare, and Novartis. CG: received speaker honoraria and travel support for attending meetings from Genzyme, Novartis, and Bayer Healthcare.

## Abbreviations

bh-SNE, Barnes-Hut Stochastic Neighbor Embedding; BBB, blood–brain-barrier; cm, central memory; CNS, central nervous system; CSF, cerebrospinal fluid; em, effector memory; FTY, fingolimod; HD, healthy donor; HBMEC, human brain microvascular endothelial cell; ILC, innate lymphoid cells; mDC, myeloid dendritic cells; MS, multiple sclerosis; NAT, natalizumab; NK, natural killer; RRMS, relapsing–remitting multiple sclerosis; PB, peripheral blood; S1P, sphingosine-1-phosphate; SPADE, spanning-tree progression analysis of density-normalized events; Th, T-helper cells; Treg, regulatory T-cells; WO, washout.
