# Supplementary material for: Immune Cell Profiling During Switching from Natalizumab to Fingolimod Reveals Differential Effects on Systemic Immune-Regulatory Networks and on Trafficking of Non-T Cell Populations into the Cerebrospinal Fluid—Results from the ToFingo Successor Study
Source: Front Immunol. 2018 Jul 9;9:1560. doi: 10.3389/fimmu.2018.01560 (PMC6052886; doi:10.3389/fimmu.2018.01560)
Supplement: Supplementary file 1 [file data_sheet_1.doc]

*Supplementary Material*

**Immune Cell Profiling During Switching from Natalizumab to Fingolimod Reveals Differential Effects on Systemic Immune-Regulatory Networks and on Trafficking of Non-T Cell Populations into the Cerebrospinal Fluid**

**– Results from the ToFingo Successor Study**

Lisa Lohmann, Claudia Janoschka, Andreas Schulte-Mecklenbeck, Svenja Klinsing, Lucienne Kirstein, Uta Hanning, Timo Wirth, Tilman Schneider-Hohendorf, Nicholas Schwab, Catharina C Gross, Maria Eveslage, Sven G Meuth, Heinz Wiendl, and Luisa Klotz*

*Corresponding author:

Prof. Dr. med Luisa Klotz, University Hospital Münster, Albert-Schweitzer-Campus 1, 48149 Münster, Germany, Email: luisa.klotz@ukmuenster.de

**Supplemental Figure S1:**

**
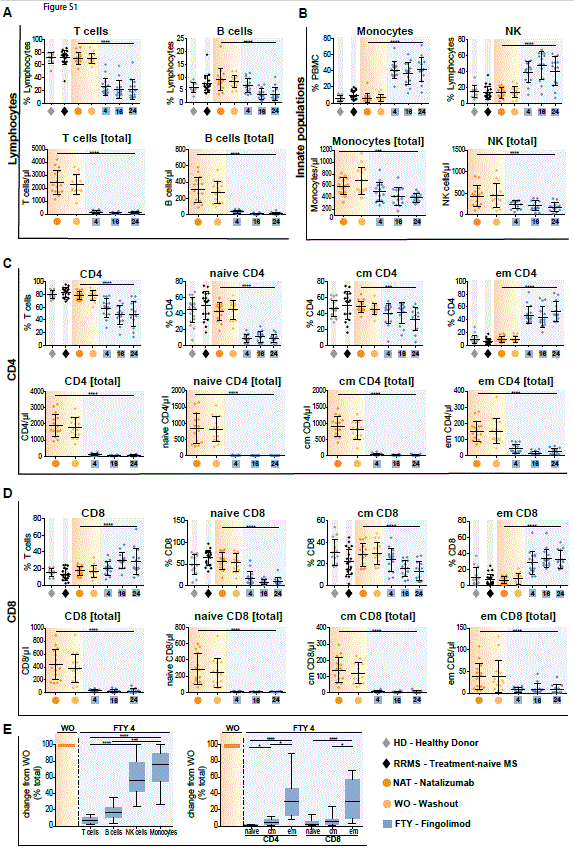
**

**Supplemental Figure S1**: **Overview of comprehensive changes of immune cell subset compositions and differential effects on pro- and anti-inflammatory T cells**. Data (n=15) was assessed at baseline under long-term natalizumab treatment (NAT; orange), after 8 weeks of washout without treatment (WO; light orange) and at time points 4 weeks (FTY4; blue), 16 weeks (FTY16; blue) and 24 weeks (FTY24; blue) after onset of fingolimod treatment. Healthy donors (HD; grey) and treatment naive RRMS patients (RRMS; black) represent control groups. (**A-D**) Flow cytometry data (n=15) represents proportional changes (in %) and total cell count (in µl) of (**A**) T cells and B cells, (**B**) monocytes and NK cells, (**C**) CD4 and CD4 subsets (**D**) CD8 and CD8 subsets at indicated time points. (**E**) Change in absolute cell counts after treatment switch by comparison of FTY4 to the WO time point (in % total) of T cells, B cells, NK cells and monocytes, as well as CD4 and CD8 subpopulations. Values represent mean ± SD. Statistical significance was evaluated by linear mixed model; Friedman test (1-way ANOVA). *P ≤ .05; ***P ≤ .001; ****P ≤ .0001.

**Supplemental Figure S2:**

**
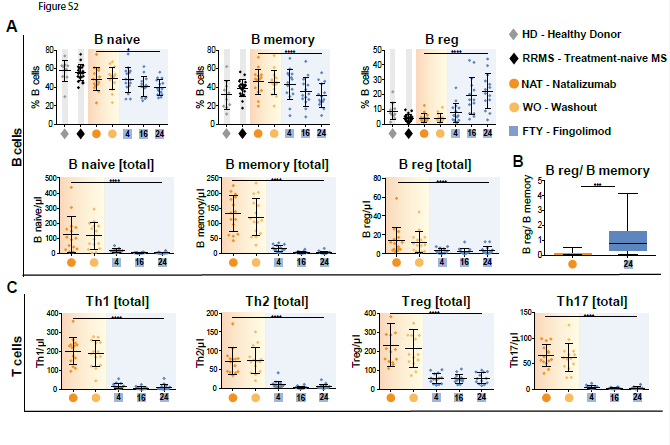
**

**Supplemental Figure S2**: **Overview of comprehensive changes of immune cell subset compositions and differential effects on pro- and anti-inflammatory T cells**. Data (n=15) was assessed at baseline under long-term natalizumab treatment (NAT; orange), after 8 weeks of washout without treatment (WO; light orange) and at time points 4 weeks (FTY4; blue), 16 weeks (FTY16; blue) and 24 weeks (FTY24; blue) after onset of fingolimod treatment. Healthy donors (HD; grey) and treatment naive RRMS patients (RRMS; black) represent control groups. (**A**) Flow cytometry data (n=15) represents proportional changes (in %) and total cell count (in µl) of (**A**) B cell subpopulations, (**B**) B reg/B memory ratio at baseline compared to study endpoint (FTY24). (**C**) Absolute counts of Th- subpopulations Th1, Th2, Treg and Th17 in µl. Values represent mean ± SD. Statistical significance was evaluated by linear mixed model; Wilcoxon matched-pairs signed rank test. *P ≤ .05; ***P ≤ .001; ****P ≤ .0001.

**Supplemental Figure S3:**

**
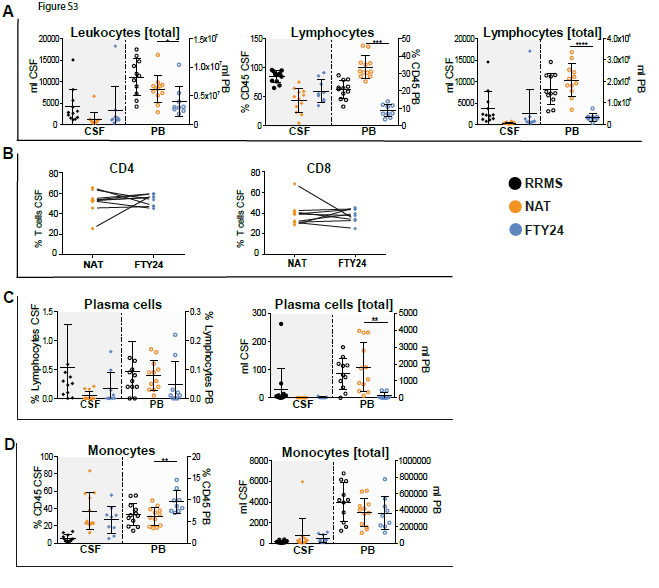
**

**Supplemental Figure S3: Natalizumab inhibits non-T cells invasion into the CSF more efficiently than fingolimod.** Assessment of flow cytometry data attained from fresh cerebrospinal fluid (CSF, closed dots) and corresponding peripheral blood (PB, open dots) samples, at baseline under long-term natalizumab treatment (NAT; n=12; orange) and 24 weeks after onset of fingolimod treatment (FTY24; n=9; blue) compared to naive MS patients (RRMS; black). (**A**) Total leukocyte and lymphocyte counts (per ml) and relative proportions (in %) of lymphocytes. (**B**) Plots display relative proportions (in %) of CD4 and CD8 cells at baseline under long-term natalizumab treatment (NAT; n=12) and 24 weeks after onset of fingolimod treatment (FTY24; n=9); black lines indicate the pairwise changes of each individual patient. Relative proportions (in %) and total cell count in ml of (**C**) plasma cells and (**D**) monocytes. Statistical significance was evaluated by linear mixed model. All values represent mean ± SD. *P ≤ .05; **P ≤ .01; ***P ≤ .001; ****P ≤ .0001.

**Supplemental Figure S4**

**
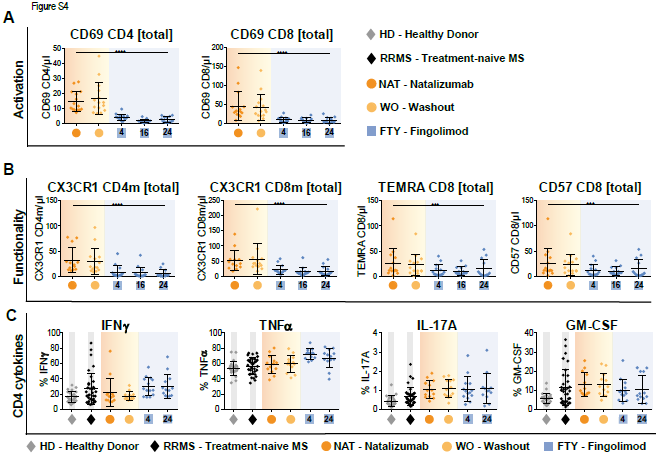
**

**Supplemental Figure S4: Preferential enrichment of terminally differentiated and functionally highly competent effector T cell subsets under fingolimod treatment.** All data (n=15) was assessed at baseline under long-term natalizumab treatment (NAT), after 8 weeks washout without treatment (WO) and at time points 4 weeks (FTY4), 16 weeks (FTY16) and 24 weeks (FTY24) after onset of fingolimod treatment. Healthy donors (HD) and treatment naive RRMS patients (RRMS) represent control groups. Absolute cell counts of terminally and functionally differentiated populations (**A**) CD69^+^CD4 and CD69^+^CD8, (**B**) CX3CR1^+^CD4, CX3CR1^+^CD8, TEMRA^+^CD8 and CD57^+^CD8. (**C**) Intracellular cytokine assay was performed to assess cytokine production; cells were stimulated with leukocyte activation cocktail (LAC) for 6h and analyzed via flow cytometry. Scatter plots show values ±SD for selected cytokines, IL-17A, GM-CSF, IFNγ and TNFα of CD4 T cells in percent, at indicated treatment time points. Statistical significance was evaluated by linear mixed model and Kruskal-Wallis test. ***P ≤ .001; ****P ≤ .0001.

**Supplemental Figure S5**

**
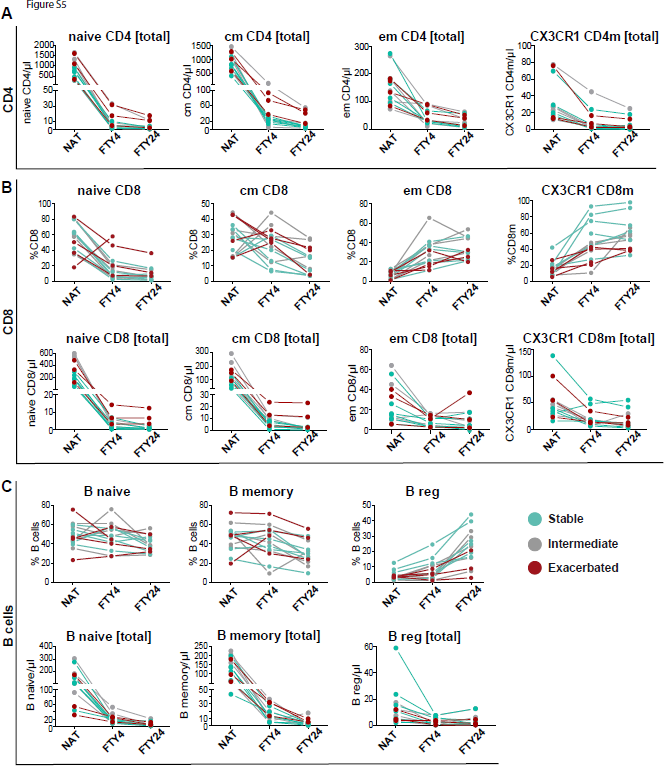
**

**Supplemental Figure S5. Patients with a stable disease course exhibit significantly reduced CD49d expression levels under long-term natalizumab treatment.** To assess correlation of peripheral immune signatures with clinical response, the study cohort was divided into subgroups: stable (blue; n=6), intermediate (grey; n=5) and exacerbated (red; n=4 time point NAT and FTY4; n=3 time point FTY24) estimated by clinical criteria. Comparison of population frequency (%) and absolute cell count (in µl) between baseline long-term natalizumab treatment (NAT), at time points 4 weeks (FTY4) and 24 weeks (FTY24) after onset of fingolimod treatment, in (**A**) CD4 T cell subsets, (**B**) CD8 T cell subsets and (**C**) in B cell subsets are shown.

**Supplemental Table S1.** Eligibility criteria.

| Inclusion criteria | - Written informed consent provided - Male and female subjects aged 18 to 65 years - Diagnosis of RRMS, defined by revised McDonald criteria (2010) - Expanded Disability Status Scale (EDSS) at screening score ≤ 6.0 - Treatment with natalizumab for at least 12 months prior to screening and consideration of treatment discontinuation for any of the following reasons:   - treatment duration > 2 years   - positive JCV antibody status   - adverse effects including hypersensitivity reactions   - presence of anti-natalizumab neutralizing antibodies   - any other valid medical reason |
| --- | --- |
| Exclusion criteria | - History of chronic disease of the immune system other than MS, which requires systemic immunosuppressive treatment, or a known immunodeficiency syndrome - Diagnosis of Crohn’s disease or ulcerative colitis - Treatment with:   - systemic corticosteroids or immunoglobulins within 1 month prior to baseline   - immunosuppressive medications such as azathioprine, cyclophosphamide or methotrexate within 3 months prior to baseline   - monoclonal antibodies (excluding natalizumab) within 3 months prior to baseline.   - cladribine or mitoxantrone at any time. - History of malignancy of any organ system (other than cutaneous basal cell carcinoma) - Uncontrolled diabetes mellitus (HbA1c >7 %) - Diagnosis of macular edema during screening phase - Severe active infections, active chronic infection - Negative for varicella-zoster virus IgG antibodies prior to baseline - Vaccination with live or live-attenuated vaccine (including varicella-zoster virus or measles) within 1 month prior to baseline - Previous therapy with total lymphoid irradiation or bone marrow transplantation - Any medically unstable condition, as assessed by the investigator - Any of the following cardiovascular conditions and/or findings in the screening ECG:   - history of cardiac arrest   - myocardial infarction within the past 6 months prior to screening or with current unstable ischemic heart disease   - history of angina pectoris due to coronary spasm   - heart failure (NYHA III-IV) or any severe cardiac disease as determined by the investigator - history or presence of a second-degree AV block, Type II or a third-degree AV block or QTc interval > 450 ms in males and > 470 ms in females - treatment with Class Ia (ajmaline, disopyramide, procainamide, quinidine) or Class III antiarrhythmic drugs (e.g., amiodarone, bretylium, sotalol, ibulitide, azimilide, dofelitide) - proven history of sick sinus syndrome or sino-atrial heart block - uncontrolled hypertension - Resting HR lower than 45 bpm - Presence of severe respiratory disease, pulmonary fibrosis or chronic obstructive pulmonary disease (Class III-IV) - Any of the following hepatic conditions: - severe hepatic injury (Child-Pugh class C) - total bilirubin greater than 2 times the upper limit of the normal range - AST or ALT greater than 2 times the upper limit of the normal range - alkaline phosphatase (AP) greater than 2 times the upper limit of the normal range - White blood cell (WBC) count <3,500/mm3 or lymphocyte count <800/mm3 at screening - Any of the following neurologic/psychiatric disorders:   - history of substance abuse (drug or alcohol) in the past five years or any other factor (i.e., serious psychiatric condition) that may interfere with the subject’s ability to cooperate and comply with the study procedures   - progressive neurological disorder, other than MS, which may affect participation in the study - Incapability to undergo MRI scans, including claustrophobia or history of hypersensitivity to gadolinium-DTPA - Treatment with investigational drug or therapy within 180 days or 5 half-lives before baseline, whichever is longer - Pregnancy or breast feeding (lactating), confirmed by a positive β-hCG laboratory test - Child-bearing potential (Unless the women concerned are using effective contraception during the study and for 5 half-lives after stopping treatment. In case of use of oral contraception women should have been stable on the same medication for a minimum of 3 months before baseline) - History of hypersensitivity to the study drugs or to drugs of similar chemical classes - Prior participation in a trial with fingolimod |

**Supplemental Table S2.** List of antibody combination for identification of cell populations

Figure 1:

| T- helper cells 1^+^ (**Th1**) | CD14^-^CD3^+^CD56^-^CD4^+^CD8^-^CD45RO^+^CD194^-^CD196^-^CD183^+^CD195^+/-^ |
| --- | --- |
| T- helper cells 2^+^ (**Th2**) | CD14^-^CD3^+^CD56^-^CD4^+^CD8^-^CD45RO^+^CD194^+^CD196^-^CD183^-^CD195^+/-^ |
| T- regulatory cells^+^ (**Treg**) | CD3^+^CD56^-^CD4^+^CD8^-^CD127lowCD25^+^ |
| T- helper cells 17^+^ (**Th17**) | CD14^-^CD3^+^CD56^-^CD4^+^CD8^-^CD45RO^+^CD194^+^CD196^+^CD146^+^CD161^+^ |

Figure 2:

| CD4^+^ T cells **(CD4)** | CD45^+^CD14^-^CD3^+^CD56^-^CD4^+^CD8^-^ |
| --- | --- |
| CD8^+^ T cells **(CD8)** | CD45^+^CD14^-^CD3^+^CD56^-^CD4^-^CD8^+^ |
| B cells ^+^ **(B cells)** | CD45^+^CD14^-^CD138^-^CD19^+^ |
| natural killer cells^+^ **(NK)** | CD45^+^CD14^-^CD3^-^CD56^+^ |
| Monocytes^+^ **(CD14^+^CD16^-^)** | CD45^+^CD14^+^CD16^-^ |
| Monocytes^+^ **(CD14^+^CD16^+^)** | CD45^+^CD14^+^CD16^+^ |

Figure 3:

| CD4^+^ T cells CD69^+^ **(CD69 CD4)** | CD14^-^CD3^+^CD56^-^CD4^+^CD8^-^CD69^+^ |
| --- | --- |
| CD8^+^ T cells CD69^+^ **(CD69 CD8)** | CD14^-^CD3^+^CD56^-^CD4^-^CD8^+^CD69^+^ |
| Memory CD4^+^ T cells CX3CR1^+^ **(CX3CR1 CD4m)** | CD3^+^CD56^-^CD4^+^CD8^-^CD45RO^+^CX3CR1^+^ |
| Memory CD8^+^ T cells CX3CR1^+^ **(CX3CR1 CD8m)** | CD3^+^CD56^-^CD4^-^CD8^+^CD45RO^+^CX3CR1^+^ |
| CD8^+^ TEMRA **(TEMRA CD8)** | CD3^+^CD56^-^CD4^-^CD8^+^CD45RA^+^CD27^-^ |
| CD8^+^ CD57^+^ **(CD57 CD8)** | CD3^+^CD56^-^CD4^-^CD8^+^CD45RA^+/-^CD57^+^ |
| T cells^+^ **( T cells)** | CD14^-^CD3^+^CD56^-^ |
| B cells^+^ **(B cells)** | CD14^-^CD19^+^CD1c^+/-^ |
| CD4^+^ T cells **(CD4)** | CD14^-^CD3^+^CD56^-^CD4^+^CD8^-^ |
| CD8^+^ T cells **(CD8)** | CD14^-^CD3^+^CD56^-^CD4^-^CD8^+^ |

Figure 4:

| naive CD4^+^ **(naive CD4)** | CD3^+^CD56^-^CD4^+^CD8^-^CD45RO^-^CD27^+^ |
| --- | --- |
| central memory CD4^+^ T cells **(cm CD4)** | CD3^+^CD56^-^CD4^+^CD8^-^CD45RO^+^CD27^+^ |
| effector memory CD4 ^+^T cells **(em CD4)** | CD3^+^CD56^-^CD4^+^CD8^-^CD45RO^+^CD27^-^ |
| Memory CD4^+^ T cells CX3CR1^+^ **(CX3CR1 CD4m)** | CD3^+^CD56^-^CD4^+^CD8^-^CD45RO^+^CX3CR1^+^ |
| CD4^+^ T cells CD49d^+^ **(CD49d CD4)** | CD3^+^CD56^-^CD4^+^CD8^-^CD49d^+^ |
| CD8^+^ T cells CD49d^+^ **(CD49d CD8)** | CD3^+^CD56^-^CD4^-^CD8^+^CD49d^+^ |

Supplemental Figure S1:

| T cells^+^ (**T cells**) | CD14^-^CD3^+^CD56^-^ |
| --- | --- |
| B cells^+^ (**B cells**) | CD14^-^CD19^+^CD1c^+/-^ |
| Monocytes^+^ (**Monocytes**) | CD14^+^CD56^-^CD16^+^ |
| NK cells^+^ (**NK**) | CD14^-^CD3^-^CD56^+^ |
| CD4^+^T cells (**CD4**) | CD3^+^CD56^-^CD4^+^CD8^-^ |
| naive CD4^+^ T cells (**naive CD4**) | CD3^+^CD56^-^CD4^+^CD8^-^CD45RO^-^CD27^+^ |
| central memory CD4^+^ T cells (**cm CD4**) | CD3^+^CD56^-^CD4^+^CD8^-^CD45RO^+-^CD27^+^ |
| effector memory CD4 ^+^T cells (**em CD4**) | CD3^+^CD56^-^CD4^+^CD8^-^CD45RO^+^CD27^-^ |
| CD8^+^T cells (**CD8**) | CD3^+^CD56^-^CD4^-^CD8^+^ |
| naive CD8^+^ T cells (**naive CD8**) | CD3^+^CD56^-^CD4^-^CD8^+^CD45RO^-^CD27^+^ |
| central memory CD8^+^ T cells (**cm CD8**) | CD3^+^CD56^-^CD4^-^CD8^+^CD45RO^+-^CD27^+^ |
| effector memory CD8 ^+^T cells (**em CD8**) | CD3^+^CD56^-^CD4^-^CD8^+^CD45RO^+^CD27^-^ |

Supplemental Figure S2:

| naive B cells^+^ (**B naive**)  memory B cells^+^ (**B memory**)  regulatory B cells^+^ (**B reg**)  T- helper cells 1^+^ (**Th1**)  T- helper cells 2^+^ (**Th2**)  T- regulatory cells^+^ (**Treg**)  T- helper cells 17^+^ (**Th17**) | CD14^-^CD19^+^CD20^+^CD38^-^CD27^-^  CD14^-^CD19^+^CD20^+^CD38^-^CD27^+^  CD14^-^CD19^+^CD20^+^CD38^+^CD27^-^  CD14^-^CD3^+^CD56^-^CD4^+^CD8^-^CD45RO^+^CD194^-^CD196^-^CD183^+^CD195^+/-^  CD14^-^CD3^+^CD56^-^CD4^+^CD8^-^CD45RO^+^CD194^+^CD196^-^CD183^-^CD195^+/-^  CD3^+^CD56^-^CD4^+^CD8^-^CD127^low^CD25^+^  CD14^-^CD3^+^CD56^-^CD4^+^CD8^-^CD45RO^+^CD194^+^CD196^+^CD146^+^CD161^+^ |
| --- | --- |

Supplemental Figure S3:

| Leukocytes^+^ (**Leukocytes**)  Lymphocytes^+^ (**Lymphocytes**)  CD4 T cells^+^ (**CD4**)  CD8 T cells^+^ (**CD8**)  plasma cells^+^ (**Plasma cells**)  monocytes^+^ (**Monocytes**) | CD45^+^  CD45^+^CD14^-^  CD45^+^CD14^-^CD3^+^CD56^-^CD4^+^CD8^-^  CD45^+^CD14^-^CD3^+^CD56^-^CD4^-^CD8^+^  CD45^+^CD14^-^CD138^+^CD19^-^  CD45^+^CD14^+^ |
| --- | --- |

Supplemental Figure S4

| CD4^+^ T cells CD69^+^ (**CD69 CD4**)  CD8^+^ T cells CD69^+^ (**CD69 CD8**)  Memory CD4^+^ T cells CX3CR1^+^ (**CX3CR1 CD4m**)  Memory CD8^+^ T cells CX3CR1^+^ (**CX3CR1 CD8m**)  CD8^+^ TEMRA (**TEMRA CD8**)  CD8^+^ CD57^+^ (**CD57 CD8**) | CD14^-^CD3^+^CD56^-^CD4^+^CD8^-^CD69^+^  CD14^-^CD3^+^CD56^-^CD4^-^CD8^+^CD69^+^  CD3^+^CD56^-^CD4^+^CD8^-^CD45RO^+^CX3CR1^+^  CD3^+^CD56^-^CD4^-^CD8^+^CD45RO^+^CX3CR1^+^  CD3^+^CD56^-^CD4^-^CD8^+^CD45RA^+^CD27^-^  CD3^+^CD56^-^CD4^-^CD8^+^CD45RA^+/-^CD57^+^ |
| --- | --- |
| Interferon-gamma (**IFNγ**) | CD3^+^CD4^+^CD8^-^ IFNγ^+^ |
| Tumor necrosis factor alpha **(TNFα)** | CD3^+^CD4^+^CD8^-^ TNFα^+^ |
| Interleukin 17A CD4^+^ T cells **(IL-17A)** | CD3^+^CD4^+^CD8^-^ IL-17A^+^ |
| Granulocyte-macrophage colony-stimulating factor CD4^+^ T cells **(GM-CSF)** | CD3^+^CD4^+^CD8^-^ GM-CSF^+^ |

Supplemental Figure S5

| naive CD4^+^ (**naive CD4**)  central memory CD4^+^ T cells (**cm CD4**)  effector memory CD4^+^ T cells (**em CD4**)  Memory CD4^+^ T cells CX3CR1^+^ (**CX3CR1 CD4m**)  naive CD8^+^ **(naive CD8)** | CD3^+^CD56^-^CD4^+^CD8^-^CD45RO^-^CD27^+^  CD3^+^CD56^-^CD4^+^CD8^-^CD45RO^+^CD27^+^  CD3^+^CD56^-^CD4^+^CD8^-^CD45RO^+^CD27^-^  CD3^+^CD56^-^CD4^+^CD8^-^CD45RO^+^CX3CR1^+^  CD3^+^CD56^-^CD4^-^CD8^+^CD45RO^-^CD27^+^ |
| --- | --- |
| central memory CD8^+^ T cells **(cm CD8)** | CD3^+^CD56^-^CD4^-^CD8^+^CD45RO^+^CD27^+^ |
| effector memory CD8 ^+^T cells **(em CD8)** | CD3^+^CD56^-^CD4^-^CD8^+^CD45RO^+^CD27^-^ |
| Memory CD8^+^ T cells CX3CR1^+^ **(CX3CR1 CD8m)** | CD3^+^CD56^-^CD4^-^CD8^+^CD45RO^+^CX3CR1^+^ |
| naive B cells^+^ **(B naive)** | CD14^-^CD19^+^CD20^+^CD38^-^CD27^-^ |
| memory B cells^+^ **(B memory)** | CD14^-^CD19^+^CD20^+^CD38^-^CD27^+^ |
| regulatory B cells^+^ **(B reg)** | CD14^-^CD19^+^CD20^+^CD38^+^CD27^-^ |
